# Evolution in Quantum Leaps: Multiple Combinatorial Transfers of HPI and Other Genetic Modules in *Enterobacteriaceae*

**DOI:** 10.1371/journal.pone.0008662

**Published:** 2010-01-13

**Authors:** Armand Paauw, Maurine A. Leverstein-van Hall, Jan Verhoef, Ad C. Fluit

**Affiliations:** Department of Medical Microbiology, University Medical Centre Utrecht, Utrecht, The Netherlands; University of Würzburg, Germany

## Abstract

Horizontal gene transfer is a key step in the evolution of *Enterobacteriaceae*. By acquiring virulence determinants of foreign origin, commensals can evolve into pathogens. In *Enterobacteriaceae*, horizontal transfer of these virulence determinants is largely dependent on transfer by plasmids, phages, genomic islands (GIs) and genomic modules (GMs). The High Pathogenicity Island (HPI) is a GI encoding virulence genes that can be transferred between different *Enterobacteriaceae*. We investigated the HPI because it was present in an *Enterobacter hormaechei* outbreak strain (EHOS). Genome sequence analysis showed that the EHOS contained an integration site for mobile elements and harbored two GIs and three putative GMs, including a new variant of the HPI (HPI-ICE*Eh1*). We demonstrate, for the first time, that combinatorial transfers of GIs and GMs between *Enterobacter cloacae* complex isolates must have occurred. Furthermore, the excision and circularization of several combinations of the GIs and GMs was demonstrated. Because of its flexibility, the multiple integration site of mobile DNA can be considered an integration hotspot (IHS) that increases the genomic plasticity of the bacterium. Multiple combinatorial transfers of diverse combinations of the HPI and other genomic elements among *Enterobacteriaceae* may accelerate the generation of new pathogenic strains.

## Introduction

The emergence of new pathogenic strains among the *Enterobacteriaceae* depends largely on the horizontal transfer of virulence determinants via mobile genetic elements such as plasmids, phages, genomic islands, and genomic modules [1]–[11]. Moreover, a single horizontal transfer event can alter the phenotypic and virulence characteristics. Thereby a normally benign organism can be transformed by a single step into a pathogen. This has been dubbed “evolution in quantum leaps” [3]. *Enterobacter hormaechei* is the most commonly isolated nosocomial pathogenic species of the *Enterobacter cloacae* complex (ECC) [12], [13]. A nationwide outbreak of a multidrug-resistant *E. hormaechei* outbreak strain (EHOS) occurred in The Netherlands [14]–[16]. This strain spread throughout hospitals, despite the adequate implementation of internationally accepted infection prevention guidelines, and caused invasive infections in more than 100 patients [16]. Epidemic strains, due to their prevalence, have a greater chance of acquiring new virulence and resistance genes [17], [18]. In a previous study, we showed that the chromosome of the EHOS contained the High Pathogenicity Island (HPI), which most likely increased virulence of the EHOS [19], [20]. This is supported by the fact that *Yersinia* spp., *Escherichia coli*, and possibly *Klebsiella pneumoniae* isolates containing the HPI are more virulent than isolates lacking this island [7], [9], [21]–[24]. The HPI segment that encodes the iron uptake and regulation system comprises 11 genes and is nearly identical in all detected isolates. Yersiniabactin is synthesized by a complex assembly line in which YbtS, YbtE, HMWP1, HMWP2, and YbtU are essential proteins [25], [26]. HMWP1 and HMWP2 (High Molecular Weight Protein 1 and 2) are 350 and 230 kDa proteins that are encoded by *irp1* and *irp2*, respectively [27], [28]. Other genes are involved in the regulation and transport of yersiniabactin, such as the *fyuA* gene, which encodes the yersiniabactin receptor.

Three basic variants of the HPI have been described. All HPI variants contain a common P4-like integrase, called *intB*, and a region that encodes for an iron uptake system followed by an AT-rich sequence. First, HPIs from *Yersinia* species with one or more IS elements at the 3′-end were described [21], [29]. Next, a putative integrative and conjugative element (ICE) with an HPI was found in *E. coli* ECOR31 (HPI-ICE*Ec1*) [6], [10]. Then, an ICE with a high similarity to HPI-ICE*Ec1* was found in a *K. pneumoniae* isolate [7]. This ICE, named HPI-ICE*Kp1*, contains the conserved part encoding the iron uptake system, a putative integrative and conjugative element and two additional genetic segments relative to HPI-ICE*Ec1*. One of these segments produces a catecholate-type siderophore and a regulator of the mucoid phenotype and the other segment contains hypothetical genes with similarity to genes found in *Nitrobacter hamburgensis*. The other segment, which is located after the HPI segment but in front of the putative ICE segment, is similar to part of a large plasmid in *K. pneumoniae*, and it contains genes responsible for DNA conjugative transfer [7]. Genes located on the ICE segment of HPI-ICE*Kp1* that are necessary for excision and conjugation are 99% identical to those from HPI-ICE*Ec1*. However, HPI-ICE*Kp1* lacks a putative helicase gene described for HPI-ICE*Ec1* [7], [10]. The integrase plays a critical role for horizontal transfer of genomic islands. Like phage integrases, it acts as a site-specific recombinase that catalyses both excision and integration [6]. *attO*, a 17 bp direct-repeat that flanks HPI-ICE*Ec1* and HPI-ICE*Kp1*, is the supposed integration site for HPI-ICE. HPI-ICE integrates by recombination at *attO*, resulting in a duplication of these sequences [7], [10]. The first described self-transferable genomic island was HPI-ICE*Kp1*. It was transferred to another strain *in vitro* via conjugation and possibly also *in vivo* to other *K. pneumoniae* isolates [7].

The aim of this study was to determine the heterogeneity of the HPI in the EHOS and its genetic relationship to HPIs in other *Enterobacteriaceae*. Therefore, whole genome sequencing was performed on the EHOS. The EHOS was found to contain a new HPI-ICE variant we termed HPI-ICE*Eh1*, with an ICE element highly similar to the ICE element of HPI-ICE*Ec1* from *E. coli* ECOR31 [10]. Furthermore, HPI-ICE*Eh1* was located in a region of the chromosome between another genomic island and three other genetic modules, which were all located next to each other. This region is an integration hotspot (IHS) of *E. hormaechei*, and the possibility of excision, circularization and transfer of the different genomic islands was investigated.

## Materials and Methods

### Bacterial Isolate and DNA Isolation

Based on the similarity between PFGE patterns, the clinical invasive isolate 05-545 was selected as a representative for the EHOS strain. The isolate was identified in 2005 in a routine clinical setting as *Enterobacter cloacae* using the Phoenix 100 automated microbiology system with version V3.22 software (Becton Dickinson Biosciences, Sparks, MD, USA). By *rpoB* and *hps60* genotyping, the isolate was identified as *E. hormaechei* (accession numbers: EU643417, EU643052) [13]. DNA was extracted using a NucleoSpin Tissue Kit (Macherey-Nagel GmbH & Co. KG, Düren, Germany) according to the manufacturer's guidelines.

### Genome Sequencing and Annotation

DNA was sequenced using 454 pyrophosphate sequencing technology with 24-fold coverage by Roche Applied Sciences (Roche Diagnostics, Penzberg, Germany). Subsequently, resulting reads were assembled in contigs, using the 454 Newbler assembler. A hypothetical order of the contigs was established using the genome of *Enterobacter* sp. 638 as a scaffold. This hypothetical order of contigs was used to concatenate them to each other with the recognition sequence (NNNNNCACACACTTAATTAATTAAGTGTGTGNNNNN) between contigs. This sequence contains a stop codon in each reading frame to prevent the formation of fusion proteins at the contig borders during automated annotation. Subsequently, the concatenated genome DNA sequences were submitted to the JCVI Annotation Service, where it was run through JCVI's prokaryotic annotation pipeline. Included in the pipeline are gene finding with Glimmer, Blast-extend-repraze (BER) searches, HMM searches, TMHMM searches, SignalP predictions, and automatic annotations from AutoAnnotate. (http://www.tigr.org/tigr-scripts/AnnotationEngine/ann_engine.cgi) Subsequently, the contig of interest was manually checked by comparing (putative) open reading frames (ORFs) with sequences deposited in GenBank via BLAST searches.

### Bacterial Isolates Used for HPI Prevalence and Genotyping

A total of 717 ECC isolates, one isolate per patient, were included. All isolates were genotyped by pulsed-field gel electrophoresis (PFGE) [14]–[16], [20]. The isolates were divided into UMCU outbreak related (UMCU-ECC) and non-UMCU outbreak related isolates (non-UMCU-ECC). The 305 UMCU-ECC isolates originated from the University Medical Center Utrecht, Utrecht, The Netherlands (UMCU). These isolates were representing the ECC population in the UMCU from 2001–2005 during the EHOS outbreak [16]. Of the isolates, 128 belonged to the EHOS clone (outbreak I) and 14 isolates represented three small outbreaks in which the patients had entirely overlapping times of hospitalization: outbreak IV (*E. cloacae* IV; N = six patients), outbreak VIII (*Enterobacter asburiae*; N = five patients), and outbreak IX (*E. hormaechei*; N = three patients).

The 412 non-UMCU-ECCs consist of 217 aminoglycoside-resistant ECC isolates obtained from 15 other Dutch clinical microbiology laboratories [16], 95 blood culture isolates obtained from 1989 through 2000 in the UMCU and 100 isolates obtained as part of a European antibiotic resistance surveillance (ENARE) study [30].

To study possible transfer of the HPI, a third group was included. This group contained HPI-positive *Enterobacteriaceae* isolates that did not fulfill the criteria of the first two groups. This third group contained 20 isolates, (four ECC, eleven *E. coli*, three *Enterobacter aerogenes*, and two *Citrobacter freundii*). The four ECC isolates were isolates with a different PFGE-type isolated from patients from whom an ECC was already included in the UMCU-ECC group. *Enterobacteriaceae* isolated from patients with an ECC during the outbreak period (2001–2005) were *E. coli* (11), *Enterobacter aerogenes* (2), and *Citrobacter freundii* (1). Furthermore, an *E. aerogenes* (10E013) and *C. freundii* (10A275) from the previously mentioned European antibiotic resistance surveillance study were included [30]. These last two isolates were previously misidentified as *E. cloacae*.

### HPI Detection

The presence of the *irp2*, *intB*, and *fyuA* genes were detected by PCR in lysates using the primers for *irp2*: Irp2-F, Irp2-R, *fyuA*: FyuA-F, FyuA-R, and *intB*: IntB-F, IntB-R. Characteristics and expected product sizes are described in Table S1. Amplification products were detected on 1.5% agarose gels with 1 µg/mL ethidium bromide visualized under UV light.

### Analysis of the High Pathogenicity Island

**Figure 1:**
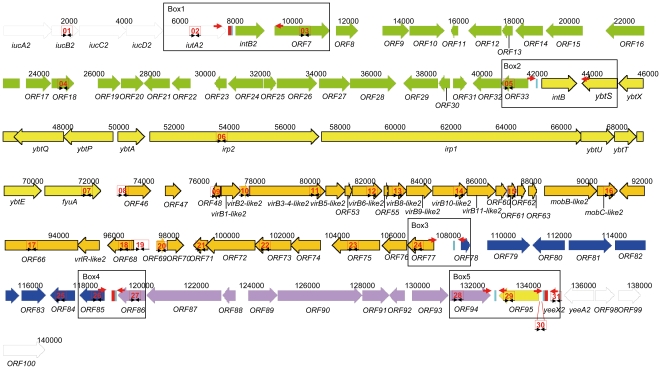
Integration hotspot with genomic islands and genetic modules of *E. hormaechei* 05-545. Accession no. FN297818. White: chromosomal DNA of *E. hormaechei* 05-545; red: *asn* tRNA; turquoise: direct repeat *attO*; green: genetic island *Eh*GI1; yellow: the conserved region of HPI-ICE*Eh1*encoding the integrase and yersiniabactin production, regulation and uptake; orange: the putative integrative and conjugative element of HPI-ICE-*Eh1*; blue: genetic module 3 (*Eh*GM3); purple: genetic module 4 (*Eh*GM4); brown: genetic module 5 (*Eh*GM5). In Box 1-5 the red arrows indicate primer positions for the linkage PCR to determine whether genomic islands are located next to each other and in which direction (see Table 3 (and Table S2) for results and Table S1 for primer characteristics). Red numbered boxes indicate positions of the amplified products by PCR for IHS characterization with primers as described in Table S1.

The contig encoding the HPI-ICE*Eh1* was screened for the presence of *attO* repeats. To investigate excision and circularization of the HPI-ICE*Eh1*, primers were designed for sites flanking all *attO* repeats (for primers see Table S1; the schematic location of the primers is depicted in Figure 1). Subsequently, PCR and nested PCR were performed on the possible remaining junctions (reformed *attO* sites) in the chromosome as well as on the extra chromosomal elements formed by the HPI-ICE*Eh1*. Obtained products were sequenced after Qiagen Quick purification (Qiagen, Westburg b.v., Leusden, The Netherlands) using the BigDye Terminator v1.3 Cycle Sequencing Ready Reaction Kit and a 3100 capillary DNA sequencer (Applied Biosystems, Nieuwerkerk a/d IJssel, The Netherlands).

### Characterization of Genomic Islands Integrated at the Integration Hotspot

To determine whether it was likely that other isolates contained a similar (putative) combination of genomic islands and genomic modules as the EHOS, several PCRs specific for the different genomic islands were performed. Primers for ICE typing were based on 17 genes described in the HPI-ICE*Ec1* present in *E. coli* ECOR31 (GenBank Accession No. AY233333, Tables S1). An additional PCR was performed for the detection of *tnpA* with primers designed based on sites adjacent to the *tnpA* gene, as *tnpA* was absent in HPI-ICE*Kp1*. Primers to type the putative genomic islands and genomic modules were based on the sequences of strain 05-545 (for primers see Table S1; the schematic location of the primers is depicted in Figure 1). To determine if the genomic islands were located adjacent to each other, linkage PCR reactions were set up using primers directed outwards from the different genomic islands and genomic modules in the IHS (Table S1, Figure 1).

### Characterization of Transferability of the HPI

To determine the genetic relationships between the conserved part of the HPI from different isolates, we extracted from GenBank all sequences with the conserved part of the HPI (the region from the *intB* gene to *fyuA* in Figure 1). Subsequently, with the 22 sequences (nine *E. coli*, two *K. pneumonia*, one *Citrobacter koseri*, two *Yersinia pseudotuberculosis*, and eight *Yersinia pestis*) and the sequence of the HPI from EHOS 05-545, a phylogenetic tree was constructed using ClonalFrame. ClonalFrame determines the genetic relationships of bacteria based on point mutations and homologous recombination [31]. The burn-in length was 50,000 iterations and Markov chain Monte Carlo iterations were set at 50,000. After every 100 iterations, a posterior sample was recorded. Finally, a majority-ruled consensus tree was generated from all posterior samples. Subsequently, the output was used to construct trees with SplitsTree4 (http://www.splitstree.org) [32]. Subsequently, congruence with the *intB* results were analyzed.

### Characterization of *intB* Genes

**Figure 2:**
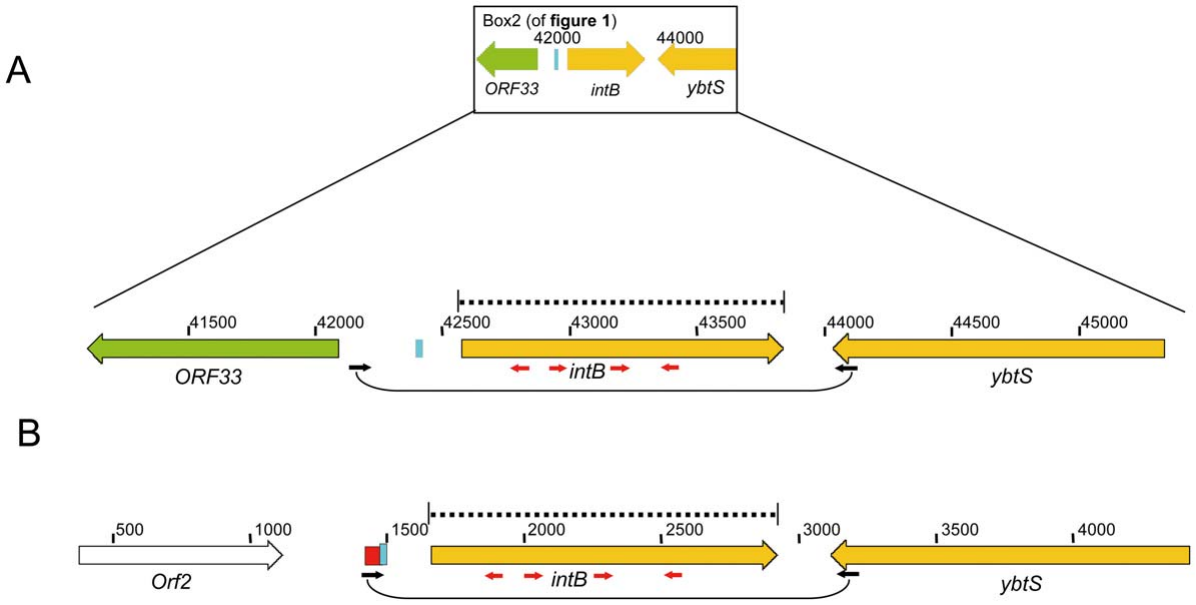
Sequencing approach for the *intB* gene of all HPI-positive isolates used in this study. A) Region bp 41,120-45,349 of FN297818 (Box 2 in Figure 1). Amplification primers (black): pre-GI1-F and YbtS-R. Sequence primers red and black for the obtained PCR product: pre-GI1-F, IntBseq2F, IntB-F, YbtS-R, IntB-R, and IntBseq1R. PCR-positive and sequenced isolates: EHOS isolates: 01-083, 01-234, 02-195, 02-203, 02-477, 03-375, 03-525, 03-577, 04-640, R1568, 05-545. Non-EHOS isolates but with the same content in the integration hotspot: 03-273, 05-349. A non-EHOS isolate with another integration hotspot: 05-316. EHOS isolates 05-761, 06-339, H9 were PCR-positive but not sequenced because ten other EHOS isolates were sequenced and did not show polymorphisms. For the other color codes see Figure 1. B) Region bp 383–4423 of AF091251. Amplification primers (black): pre-IntB-F and YbtS-R. Sequence primers red and black for the obtained PCR product: pre-IntB-F, IntBseq2F, IntB-F, YbtS-R, IntB-R, and IntBseq1R. PCR-positive and sequenced isolates: 01-084, 01-306, 02-023, 02-272, 03-018, 03-339, 03-426, 03-595, 03-613, 03-635, 03-642, 03-739, 03-895, 05-189, 05-202, 05-680, 06-316, 10A275, 10E013, 14A001, 18D099, and X2327. From the isolates 03-093, 03-192, 03-414, and R0332 the amplification product was approximately 350 bp smaller than expected. Sequence results indicated that these isolates contained a truncated *intB* gene. Moreover, bp 124–470 was deleted compared to the wild-type *intB* gene.

To obtain additional evidence on whether different isolates carried identical HPIs, the *intB* genes of 40 *irp2*-positive isolates were sequenced. The *intB* gene of isolate Q2447 was not sequenced because PCR amplification failed. Additionally, the *intB* genes of three EHOS isolates were not sequenced because 11 *intB* gene sequences were already determined from the EHOS isolates. The complete gene was amplified by two sets of specific primers. (See primers sets used for amplification and sequencing the *intB* gene of HPI (HPI-ICE*Eh1*) in Table S1, and Figure 2). Sequencing of purified PCR products was performed as described above. The resulting data were combined with 22 complete *intB* sequences present in GenBank. To test whether the phylogenetic information was biased by selection pressure, neutrality tests for selection (Tajima's D and Fu's F test) were performed using DNAsp, version 4.20.2 [33]–[35]. Subsequently, a phylogenetic tree was constructed using a Neighbor-Joining algorithm and 10,000 bootstrap iterations with Mega4.0 [36].

Furthermore, to put our data in a larger context, we constructed a phylogenetic tree of a 792 bp fragment of *intB*, previously also used for phylogenetic analysis by Schubert *et al*., and we combined the *intB* sequences from that study with our results.

### Yersiniabactin Green Fluorescent Protein (GFP) Reporter Assay

To test the expression of the iron uptake system of the HPI, yersiniabactin production was tested using a GFP-reporter assay [37]–[39]. The presence of yersiniabactin in the medium can be determined by increased production of GFP, because 267 amino acids of FyuA are fused with GFP and FyuA is upregulated in the presence of yersiniabactin. The knockout strain *Yersinia enterocolitica* WA-CS *irp1*::Kan^r^ containing the pCJG3.3N plasmid, kindly provided by Prof. J. Heesemann and Dr. S. Schubert, was used as the reporter strain to detect yersiniabactin in supernatants.

Tested isolates were cultured for seven days at 37°C under continuous shaking at 150 rpm in either 10 ml Nutrient Broth (NB), Difco nutrient broth (Becton Dickinson, Sparks, MD, USA) with 85.6 mmol NaCl or 10 ml NB with 200 µM α,α'-dipyridyl (NBD). Filter-sterilized supernatant from a culture (450 µl) was added to 50 µl of a culture of the reporter strain WA-CS *irp1*::Kan^r^ containing the pCJG3.3N plasmid. Moreover, this culture was grown overnight at 28°C in 10 ml NB with 200 µM α,α'-dipyridyl (NBD). Subsequently, the culture was centrifuged and diluted to an optical density of 0.1 at 660 nm. When the added supernatant contains yersiniabactin it will upregulate GFP production because the *fyuA* part fused to the *gfp*-gene is activated by yersiniabactin. The cultures with the GFP-strain were incubated at 28°C overnight, washed and diluted in phosphate buffered saline (PBS). The bacterial associated fluorescence and scatter data for 50,000 gated bacteria were measured using a flow cytometer (FACSCalibur, Becton Dickinson, Franklin Lanes, NJ, USA), and the mean fluorescence was determined.

### HMWP1 and HMWP 2 Expression

To test the functionality of the HPI iron uptake system, the expression of HMWP1 and HMWP2 were monitored under various conditions using SDS-PAGE. Bacteria were grown in M9 minimal medium containing 60 mM Na_2_HPO_4_, 22 mM KH_2_PO_4_, 8.6 mM NaCl, 2 mM MgSO_4_, 0.1 mM CaCl_2_, 2 g/L glucose, and 5 g/L casamino acids (pH 7.4) (M9) and in iron-depleted M9. Iron was depleted with 1% Chelex-100 for 48 h at 37°C followed by the addition of 0.5 mM α,α'-dipyridyl (Sigma-Aldrich). Proteins were extracted using sonication followed by centrifugation at 50,000×*g* for 1 h at 4°C. The pellet was incubated for 1 h in 2 mL 1% SDS and then centrifuged at 50,000×*g* for 1 h at 4°C [40]. Subsequently, the supernatant was concentrated using a Centricon YM-100 centrifugal filter (Millipore, Billerica, MA, US). Proteins were separated using 7.5% SDS-PAGE at 40 mA for 1 h, as described by Laemmli et al. [41]. The gel was stained with Coomassie Blue R. After staining, HMWP1 and HMWP2 were identified by their exceptionally large size, 350 and 230 kDa, respectively. Next, to confirm the identity of HMWP2, the proteins in the gel were electro-blotted onto an Immobilon-p Transfer Membrane (Millipore). The membrane was stained with Coomassie Blue R, and the suspected HMWP2 band was cut out and sequenced using Edman degradation (the Sequence Center, Utrecht, The Netherlands).

## Results

### Analysis of the HPI Region

HPI-ICE*Eh1* was located on one contig (accession number: FN297818). Six *attO* sites were found in this contig (Table 1). The six direct repeats are an indication that this region of 127 kb putatively contains two genomic islands and three genomic modules (Figure 1). The first genomic module is likely a genomic island because it contains all the structural features of a genomic island. (i) it is integrated at a tRNA gene (*asn* tRNA); (ii) it carries a gene for a phage type integrase; (iii) its GC-content (47.9%) is different from that of the genome (55.4%); (iv) it is flanked by 17-bp perfect direct repeats; and (v) it contains genes that possibly increase the fitness of the bacteria. This first putative genomic island of 34.2 kb (*Enterobacter hormaechei* Genomic Island 1 or *Eh*GI1) comprises 28 open reading frames (orfs) that showed similarity with a comparable region in *C. koseri* ATCC BAA-895 and partly with a DNA region in *E. coli* (ECOR31, accession no. EU681267). Only for one *orf* that encodes a putative truncated *intB2* integrase is the function clear. This *intB2* had a sequence similarity of 95% with the *intB* of HPI-ICE*Eh1*. However, it contains sequence mutations that likely lead to a truncated protein making it unlikely that this protein is functional. The remaining 27 ORFs had low similarities with known sequences. However, based on sequence comparisons, at least a part of these ORFs may encode genes involved in iron transport because a putative Fe^3+^-ABC transporter periplasmic-binding protein, a putative Fe^2+^/Zn^2+^ uptake regulation protein, a putative Fe^2+^ transport protein and an ATP-binding component of an iron transport protein were identified.

**Table 1:** 17-bp direct-repeat sequences and flanking sequences in the chromosome.

Genetic element	Sequence of recombination site		
Direct repeat on the chromosome^a^	flanking sequence	*attO*	flanking sequence
1	cgtatgtcactggttcgagt	CCAGTCAGAGGAGCCAA	Tttgctgttttcatgcatcc
2	taccaggtcgggcgtctgtg	CCAGTCAGAGGAGCCAA	ttttctgttttcatgcttcc
3	agttactggcaaaggcgatc	CCAGTCAGAGGAGCCAA	atttgaaaagcctgctttta
4	cgtatgtcactggttcgagt	CCAGTCAGAGGAGCCAA	atttgaaaagcctgctttta
5 (RC)^b^	cgtatgtcactggttcgagt	CCAGTCAGAGGAGCCAA	atttgaaaagcctgctttta
6	cgtatgtcactggttcgagt	CCAGTCAGAGGAGCCAA	atttaaaaagcctgctttta

a See Figure 2 for the location of the direct repeat in the chromosome.

b RC reverse complement of original sequence.

The second genomic module is also a genomic island because it also contains most of the structural features of a genomic island: (i) it carries a gene for a phage type integrase; (ii) it has a GC-content (52.4%) that is different from that of the genome (55.4%); (iii) it is flanked by 17-bp perfect direct repeats; and (iv) it contains genes that increase the virulence of bacteria. Although this genomic island is not directly integrated at a tRNA gene (Figure 1), homologues of this genomic island described in other *Enterobacteriaceae* are. This second genomic island, or HPI-ICE*Eh1*, is 66.2 kb and consists of two segments. The first segment, a conserved part including *intB* and the 11 genes of the yersiniabactin iron uptake system, is known as HPI. This conserved sequence is 99% identical to sequenced HPIs from *Yersinia* species, *E. coli* isolates and a *K. pneumoniae* isolate. The second segment contains the variable part of HPI-ICE*Eh1*. This segment is possibly involved in transfer of HPI-ICE*Eh1* and is 99% identical with that of HPI-ICE*Ec1*. Starting from the *fyuA* gene, the first difference with this ICE is a hypothetical protein, which is likely to be transcribed in *E. hormaechei* 05-545 but not in the ECOR31 because of a 1-bp deletion. A second 1-bp deletion in ECOR31 compared to 05-545 leads to the lack of an ORF in ECOR31 that is present in the EHOS (ORF60). Furthermore, the insertion element IS*630* found in ICE*Ec1* encoding the transposase TnpA is lacking in the ICE of HPI-ICE*Eh1.* The ICE element is necessary for the conjugation and integration of HPI [7]. In addition, ORF21a in ECOR31 is 33 amino acids smaller than its counterpart in the EHOS. Finally, ORF23 and ORF28 differ in their N-terminal regions compared to their counterparts in EHOS, ORF72 and ORF77, respectively.

The third part, *Eh*GM3, is a genomic module and possibly a genomic island. The GC-content of *Eh*GM3 (48.0%) is different from the genome. The module is flanked by 17-bp perfect direct repeats and the 3′-end is flanked by an *asn* tRNA. Although this genomic module does not contain a functional or cryptic gene encoding an integrase, it does contain an ORF that encodes a putative holin protein related to that of prophage CP-933X (51% amino acid similarity) and another ORF that encodes Tsx, a nucleoside-specific channel-forming membrane protein that also functions as a receptor for phages. Two transcriptional regulators are located in this region: a putative LysR-type and a nitrogen assimilation transcriptional regulator. *Eh*GM3 is 10.9 kb and contains in total eight ORFs.

The fourth genomic module, *Eh*GM4, is 13.7 kb and contains nine orfs, and it has a GC-content (56.3%) that is slightly different from that of the genome. It is flanked by perfect direct repeats (one orientated in reverse orientation) and is integrated between two *asn* tRNAs, of which one in the reverse orientation. Furthermore, it has no integrases or genes involved in conjugation. Most of the genes are associated with transport, including genes that encode for putative AcrA- and AcrB-like proteins. The Resistance Nodulation cell Division (RND) family-type transporter AcrB and the periplasmic accessory protein AcrA, together with the outer membrane factor TolC, form the tripartite efflux pump.

The fifth genomic module (*Eh*GM5) is 1.9 kb and contains a gene encoding a putative MATE family transport protein. The GC-content of this module is 54.2%; because of the variability of the GC-content in the genome and because of the small size of the module, this is not significantly different. However, this region is flanked by perfect direct repeats (one orientated in reverse orientation), and it is integrated between two *asn* tRNAs, of which one is in the reverse orientation. The genetic modules *Eh*GM3, *Eh*GM4, and *Eh*GM5 may be remnants of genomic islands, but they are now co-transferred by the HPI-ICE*Eh1*.

### Analysis of Integration Hotspots

**Figure 3:**
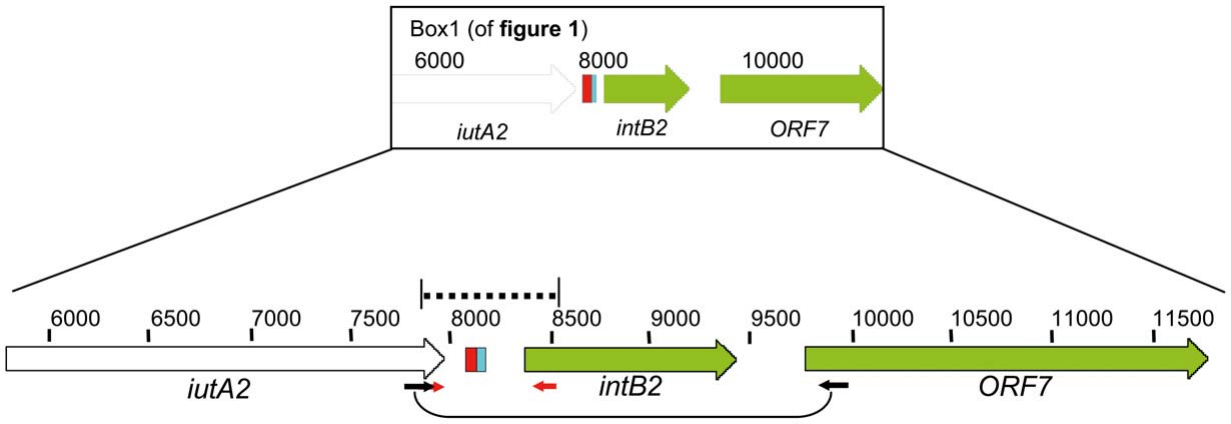
Sequence strategy for the extreme right side of the integration hotspot. PCR amplification was performed on isolates with *Eh*GI-1 used in this study. Using a PCR amplification reaction with the primers IHS-iutA-F and ORF7-2R, a product was obtained only from the 12 tested EHOS isolates and isolates 03-273 and 05-349. The amplified products were partly sequenced with primers Rev-IntB-GI1-out and For-iutA-tRNA-seq. Accession no. GQ891736-GQ891749.

The *Eh*GI1 in isolate 05-545 is integrated at an *asn* tRNA after the *iutA2* gene in the chromosome. By sequencing this region in 12 EHOSs and the two ECCs we showed that the integration site was identical in all these isolates (Figure 3, Accession no. GQ891736-GQ891749). The IHS was located after the same *asn* tRNA, indicating that the IHS, at least in the EHOS, was always located after *iutA2*.

### Excision and Circularization of the Genomic Islands in the HPI Region

Because six direct repeats and two integrases (of which *intB2* is almost certainly truncated) were identified, the possibility of various excision events and extrachromosomal circularizations in isolate 05-545 was investigated. Four different circular extrachromosomal elements were detected by PCR. These consisted of *Eh*GI1 - HPI-ICE*Eh1* (Figure S1), *Eh*GI1 - HPI-ICE*Eh1* - *Eh*GM3 (Figure S2), HPI-ICE*Eh1* (Figure S3) and HPI-ICE*Eh1* - *Eh*GM3 (Figure S4). The *attO* sequence was present in all these extrachromosomal elements (Accession no: GU086403, FN556610- FN556612). Other combinations were not detected but could not be excluded. The junction in the chromosome formed after excision of *Eh*GI1 and HPI-ICE*Eh1* could also be detected (Figure S5A). After this excision, *Eh*GM3, *Eh*GM4 and *Eh*GM5 were still in the chromosome. The junction contained the *attO* site (FN556613). In the chromosome of an HPI-negative EHOS isolate (03-638), a junction containing the *attO* site was also detected with *Eh*GM3, *Eh*GM4, and *Eh*GM5 in the chromosome. Furthermore, there was also an indication that the junction containing the *attO* site and only *Eh*GM4 and *Eh*GM5 were present in the chromosome of EHOS 05-545 and 03-638 (Figure S5B). However, this was not confirmed by sequencing because of the small amount of products obtained after the amplification reaction.

### Presence of the HPI in Other *Enterobacteriaceae*

PCR amplification of the *intB, irp2* and *fyuA* genes was performed for 137 ECC isolates to determine which gene was the most representative for the presence of the HPI. The isolates were composed of a subset of UMCU-ECCs and included 59 EHOSs and 78 other isolates. All three PCRs were positive for 57 (56 EHOS) isolates, and these were considered HPI-positive. The *intB* PCR was also positive for six additional isolates, making the *intB* PCR unreliable for HPI detection. Possibly, these six *intB*-positive samples contained *Eh*GI1 with the highly similar *IntB2* gene or P4 phages that also have an integrase gene similar to *intB*. From the *irp2* and *fyuA* genes, *irp2* was arbitrarily chosen as the marker used to test the remaining isolates for the presence of HPI [42].

Based on the *irp2* PCR, the HPI was present in 92% (184/199) of the isolates belonging to the EHOS and 2.1% (11/518) of all other ECC tested isolates. During the outbreak, EHOS isolates with and without the complete HPI-ICE*Eh1* were retrieved from at least four patients (data not shown), and 8% of all examined EHOS isolates did not contain the HPI, which is an indication that HPI is often lost in EHOS isolates. Six of the 12 HPI- negative EHOS isolates of which the origin of isolation could be determined were isolated from urine samples. Since yersiniabactin is unstable under acidic conditions, excision of the HPI may confer a fitness advantage to the bacteria in acidic environments [43]. The strains involved in the small outbreaks IV, VIII and IX were HPI-negative. The 4.0% (7/176) HPI-positive isolates in the non-EHOS isolates of the UMCU-ECC group was significantly higher (p<0.05) than the 1.2% (4/342) prevalence in the non-UMCU-ECC group. This suggests that in the UMCU during the outbreak, HPI-positive isolates were selected or that horizontal transfer of HPI occurred *in vivo* in the UMCU during the outbreak period (Table 2).

**Table 2:** Prevalence of the HPI in the EHOS and other ECC isolates in groups of different origins.

		EHOS		Non-EHOS	
Origin	Total no. of isolates	n	HPI pos. (%)	n	HPI pos. (%)
UMCU-ECC	305	129	123 (95)	176	7 (4.0)^a^
Non-UMCU-ECC	412	70	61 (87)	342	4 (1.2)^a^

a Fisher exact test (p<0.05).

### Characterization of the ICE Region of the HPI

The presence of genes possibly involved in the transfer of HPI-ICE*Eh1* were investigated because in previous studies the ICE segment of the HPI-ICE was shown to be necessary for the autonomic integration and circularization of the HPI [7], [10]. The presence of the 17 different genes known to be located on HPI-ICE*Ec1* was assessed in 44 *irp2*-positive isolates. All 14 *irp2*-positive non-EHOS isolates were selected, as were 14 EHOS isolates, 11 *E. coli*, 2 *C. freundii*, and 3 *E. aerogenes*. (Table 3 and Table S2). Thirteen *irp2*-negative ECC isolates, including two HPI-negative EHOS isolates, served as negative controls. All 14 EHOS isolates had an ICE region that was identical to the ICE of *E. coli* ECOR31, with the exception that the EHOS ICE region lacked the *tnpA* gene, similar to HPI-ICE*Kp1* (Table 3 and Table S2). The HPI-ICEs of five other ECC isolates were identical to that of the EHOS. Four of the five were related to the outbreak in the UMCU (03-273, 05-349, 03-635 and 03-739). One isolate (14A001) was obtained in Poland. Of the 11 HPI-negative control isolates, 10 lacked all HPI-ICE genes tested, while one isolate was positive for *orf19*. Since *orf19* was also present in four other isolates (two ECC and two *E. coli*), we assume that the primers selected to detect *orf19* lacked sufficient specificity or that a copy of this gene is present elsewhere in these isolates.

**Table 3:** Prevalence, localization and partial characterization of the genomic islands located in the integration hotspot of the EHOS.

Genomic island/module				1		2		3		4		5			
Isolates	Chromosome	Chromosome	Linkage PCR 1	*Eh*1-GI1-2	*Eh*1-GI1-12	*Eh*1-GI1-26	Linkage PCR 2	*Irp2*	*FyuA*	*Na-FuyA*	*Orf0*	*pilX2*	*pilX3,4*	*pilX6*	*pilX8*	*pilX10*	*YggA*	*MobC*	*Orf17*	*Orf19*	*TnpA-like*	*Orf21a-b*	*VC0181*	*VC0179*	*Orf26*	*Orf28*	Linkage PCR 3	*Eh*1-GI3-02	*Eh*1-GI3-07	Linkage PCR 4	*Eh*1-GI4-01	*Eh*1-GI4-08	Linkage PCR 5	*Eh*1-GI5-01	*Eh*1-GI5-ncr	Linkage PCR 6	Chromosome	Chromosome
HPI-core positive																																						
*E. coli* (ECOR31)								+	+	+	+	+	+	+	+	+	+	+	+	+	+^a^	+	+	+	+	+												
EHOS (14)	+	+	+	+	+	+	+	+	+	+	+	+	+	+	+	+	+	+	+	+	+^b^	+	+	+	+	+	+	+	+	+	+	+	+	+	+	+	+	+
ECC (2)	+	+	+	+	+	+	+	+	+	+	+	+	+	+	+	+	+	+	+	+	+^b^	+	+	+	+	+	+	+	+	+	+	+	+	+	+	+	+	+
ECC (2)	+	+	−	−	−	−	−	+	+	+	+	+	+	+	+	+	+	+	+	+	+^b^	+	+	+	+	+	−	+	+	−	+	+	−	+	+	+	+	+
ECC (1)	+	+	−	−	−	−	−	+	+	+	+	+	+	+	+	+	+	+	+	+	+^b^	+	+	+	+	+	−	−	−	−	−	−	−	+	+	−	+	+
ECC (1)	−	−	−	−	−	−	−	+	+	+	+	+	+	+	+	+	+	−	−	−	−	−	−	−	−	−	−	−	−	−	−	−	−	−	−	−	−	−
ECC (1)	+	+	−	−	−	−	−	+	+	+	+	+	+	−	+	+	−	−	−	+	−	−	−	−	−	−	−	+	+	+	+	+	−	+	−	−	+	+
ECC (1)	−	−	−	−	−	−	−	+	+	+	−	−	−	−	−	−	−	−	−	−	−	−	−	−	−	−	−	−	−	−	−	−	−	−	−	−	−	+
ECC (1)	−	−	−	−	−	−	−	+	+	+	−	−	−	−	−	−	−	−	−	−	−	−	−	−	−	−	−	−	−	−	−	−	−	−	−	−	−	−
ECC (1)	−	−	−	−	−	−	−	+	+	+	−	−	−	−	−	−	−	−	−	−	−	−	−	−	−	−	−	−	−	−	−	−	−	−	−	−	+	+
ECC (1)	−	−	−	+	+	+	−	+	+	+	−	−	−	−	−	−	−	−	−	−	−	−	−	−	−	−	−	−	−	−	−	−	−	−	−	−	−	−
ECC (1)	−	−	−	−	−	−	−	+	+	−	−	−	−	−	−	−	−	−	−	−	−	−	−	−	−	−	−	−	−	−	−	−	−	−	−	−	−	−
ECC (1)	+	+	−	−	−	−	−	+	+	−	−	−	−	−	−	−	−	−	−	−	−	−	−	−	−	−	−	+	+	+	+	+	+	+	+	+	+	+
ECC (1)	−	+	−	−	−	−	−	+	+	−	−	−	−	−	−	−	−	−	−	+	−	−	−	−	−	−	−	−	−	−	−	+	−	−	−	−	+	+
*C. freundii* (2)	−	−	−	−	−	−	−	+	+	+	−	−	−	−	−	−	−	−	−	−	−	−	−	−	−	−	−	−	−	−	−	−	−	−	−	−	−	−
*E. aerogenes* (3)	−	−	−	+	+	+	−	+	+	+	+	+	+	+	+	+	−	−	−	−	−	−	−	−	−	−	−	−	−	−	−	−	−	−	−	−	−	−
*E.coli* (7)	−	−	−	−	−	−	−	+	+	+	−	−	−	−	−	−	−	−	−	−	−	−	−	−	−	−	−	−	−	−	−	−	−	−	−	−	−	−
*E. coli* (2)	−	−	−	−	−	−	−	+	+	+	+	+	+	+	+	+	+	−	−	−	−	−	−	−	−	−	−	−	−	−	−	−	−	−	−	−	−	−
*E. coli* (1)	−	−	−	−	−	−	−	+	+	−	−	−	−	−	−	−	−	−	−	−	−	−	−	−	−	−	−	−	−	−	−	−	−	−	−	−	−	−
*E. coli* (1)	−	−	−	−	−	−	−	+	+	+	+	+	+	+	+	+	+	−	−	+	−	−	−	−	−	−	−	−	−	−	−	−	−	−	−	−	−	−
HPI-core negative																																						
EHOS (1)	+	+	−	+	+	+	−	−	−	−	−	−	−	−	−	−	−	−	−	−	−	−	−	−	−	−	−	+	+	+	+	+	+	+	+	+	+	+
EHOS (1)	+	+	−	−	−	−	−	−	−	−	−	−	−	−	−	−	−	−	−	−	−	−	−	−	−	−	−	+	+	+	+	+	+	+	+	+	+	+
ECC (1)	+	+	−	−	−	−	−	−	−	−	−	−	−	−	−	−	−	−	−	−	−	−	−	−	−	−	−	+	+	−	+	+	+	+	+	+	+	+
ECC (1)	+	+	−	−	−	−	−	−	−	−	−	−	−	−	−	−	−	−	−	−	−	−	−	−	−	−	−	+	+	−	+	+	−	+	+	+	+	+
ECC (1)	−	+	−	−	−	−	−	−	−	−	−	−	−	−	−	−	−	−	−	−	−	−	−	−	−	−	−	−	−	−	−	−	−	−	−	−	+	+
ECC (2)	+	+	−	−	−	−	−	−	−	−	−	−	−	−	−	−	−	−	−	−	−	−	−	−	−	−	−	+	+	+	+	+	+	+	+	+	+	+
ECC (1)	−	+	−	−	−	−	−	−	−	−	−	−	−	−	−	−	−	−	−	−	−	−	−	−	−	−	−	+	+	−	−	−	−	−	−	−	+	+
ECC (4)	+	+	−	−	−	−	−	−	−	−	−	−	−	−	−	−	−	−	−	−	−	−	−	−	−	−	−	+	+	−	+	+	−	+	+	+	+	+
ECC (1)	+	+	−	−	−	−	−	−	−	−	−	−	−	−	−	−	−	−	−	+	−	−	−	−	−	−	−	+	+	+	+	+	+	+	+	+	+	+

Genes, open reading frames (ORFs) and DNA sequences known to be located on genomic islands of the EHOS. “+” represents that a product was amplified and “−” represents a negative result for the different DNA fragments investigated (results are depicted in more detail in Table S2).

a Fragment size 1767 bp; orf20 present.

b Fragment size 612 bp; orf20 absent.

c possible positive result caused by cross-hybridization with the *intB* of the HPI.

### Stability of the Other Genomic Islands and Genomic Modules in the EHOS

To test for the presence of other genomic islands and/or genomic modules in the EHOS, PCRs 1 to 32 and the five linkage PCRs used for the characterization of the IHS (Table S1) were also performed on 16 EHOS isolates (14 HPI-positive and 2 HPI-negative, Table 3, Table S2). All 14 *irp2*-positive EHOS isolates contained (besides the HPI) the genomic island *Eh*GI1 and the three putative genomic modules in the same order. Of the two HPI-negative EHOS isolates tested, one (03-797) harbored the genomic modules *Eh*GM3, *Eh*GM4, and *Eh*GM5. The other HPI-negative EHOS (03-638) contains *Eh*GI1 in addition to the genomic modules *Eh*GM3, *Eh*GM4, and *Eh*GM5. *Eh*GI1 was apparently located on another region of the genome because no product was obtained with the linkage PCR from *Eh*GI1 to the expected chromosomal border of *Eh*GI1. However, *Eh*GM3, *Eh*GM4, and *Eh*GM5 were located in the same IHS because with a linkage PCR we could show that *Eh*GM3 was located after the *asn* tRNA behind the *iutA*2 gene. This shows that *Eh*GM3, *Eh*GM4, and *Eh*GM5 were integrated at the same IHS. Furthermore, in this isolate a junction containing the *attO* site with only *Eh*GM4 and *Eh*GM5 was also detected. The two combinations of genomic islands in this isolate indicate the flexibility of the IHS in isolate 03-638.

To determine if the same structure of the genomic islands and genomic modules present in the EHOS were also present in the other 41 isolates, PCRs were performed to characterize the IHS region of the HPI of these isolates (Table 3, Table S2). Two of the five ECC isolates with the same HPI-ICE*Ec1* were PCR-positive for the genomic island/module-specific PCRs and linkage PCRs (isolates 03-273 and 05-349). This indicated that in these two isolates, *Eh*GI1 through *Eh*GM5 were present and located in the same order and orientation as in the EHOS. Two isolates with a similar HPI-ICE structure to the EHOS did not contain *Eh*GI1, but contained *Eh*GM3, *Eh*GM4 and *Eh*GM5 (isolates 03-739 and 03-635). One isolate contained *Eh*GM5 next to the HPI-ICE (isolate 14A001). However, linkage PCRs showed that in this 14A001 isolate, *Eh*GM5 and HPI-ICE were apparently not located next to each other. Furthermore, different combinations of the genomic islands/modules were found in HPI-positive and HPI-negative isolates, and these genomic islands/modules were not always located next to each other based on the results of the linkage PCRs. All HPI-positive *E. coli* and *C. freundii* tested contained none of the other genomic islands except the HPI. All three *E. aerogenes* and one ECC isolate contained *Eh*GI1, but it was likely at a different chromosomal location than the HPI because linkage PCRs from *Eh*GI1 to the HPI were all negative.

### Phylogenetic Analysis of the Conserved Part of the HPI

**Figure 4:**
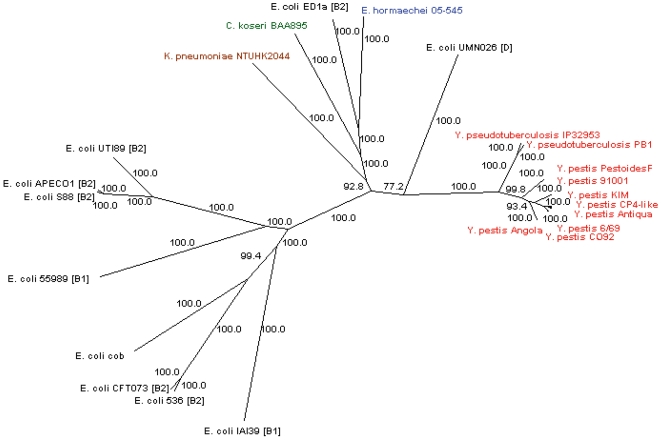
Phylogenetic tree based on the sequence of the conservative part of the HPI-ICE of 23 *Enterobacteriaceae*. The phylogenetic tree is based on the sequences of the conservative part of the HPI-ICE (homologous to bp 42589-72874 of accession no. FN297818) of 23 *Enterobacteriaceae* clustered with Clonalframe. Numbers indicate confidence values of the branches. The phylogenetic group membership of the *E. coli* strain is indicated between brackets.

To determine the genetic relationships between the HPI-ICE in EHOS 05-545 and 22 other HPI-ICEs in other *Enterobacteriaceae*, ClonalFrame was used. Therefore, the conserved regions of HPI-ICEs (homologies of sequences bp 42,589–72,874 of FN297818 bp) encoding the integrase and yersiniabactin production, regulation and uptake genes were used. The recombination/mutation ratio was 0.740 (95% confidence interval 0.414–1.250), indicating that the mutation rate is influencing the evolution of the conserved region of the HPI more than recombination events. The phylogenetic tree based on the conserved region of the HPI-ICEs shows that the conserved region of the HPIs of *Y. pseudotuberculosis* and *Y. pestis* isolates are highly related to each other. This indicates that the conserved region of the HPI in *Yersinia* is highly stable and not subjected to recombination events. These HPIs most probably descend from a single ancestor (Figure 4). In contrast to the low genetic diversity in *Yersinia* species, there is great genetic diversity in the conserved HPI-ICE regions of *E. coli* isolates. Eight *E. coli* formed a separate, but broad, branch while two *E. coli* clustered separately. *E. coli* UMN026 clustered with none of the other HPI-ICE regions, while the HPI-ICE region of *E. coli* ED1a clustered with the HPI-ICE regions of *E. hormachei* 05-545 (EHOS), *C. koseri* BAA-895, and *K. pneumoniae* NTUHK2044. Touchon *et al.* constructed a phylogenetic tree of 20 *E. coli* and *Shigella* strains based on 1,878 genes of the *E. coli* core genome [44]. This tree showed that *E. coli* ED1a is genetically most related to *E. coli*'s CFT073, 536, S88, APEC01, and UTI89, which are all strains of phylogenetic group B2. However, as mentioned the conserved HPI-ICE region from *E. coli* AD1a is not related to the conserved HPI-ICE regions of the other *E. coli*. These results and the different HPI-ICE types found in different *E. coli* strains indicate that multiple transfers of the conserved region of the HPI-ICE into *E. coli* isolates must have occurred.

The branch with *E. coli* ED1a contains four isolates that belong to different species. As mentioned, the HPI-ICE of *K. pneumoniae* has been transferred to other species [7]. Furthermore, we showed that ECOR31 and two ECC isolates also contained a highly similar HPI-ICE. BLAST analysis of the ICE segment of HPI-ICE (the variable region of the HPI) of ECOR31 showed that it is most similar to the HPI-ICE of 05-545. However, *E. coli* ED1a, *C. koseri* BAA-895, *K. pneumoniae* NTUHK2044, and *E. coli* UMN026 also contain segments highly similar to the integrative and conjugative part of the HPI of ECOR31 and EHOS 05-545, while no other HPI-ICE-containing isolates annotated in GenBank contained genes that may be involved in conjugation and transfer. This result indicates that the branch with multiple species contains mostly HPI-ICEs that may be transferred. This implies that multiple species are able to transfer their HPI.

**Figure 5:**
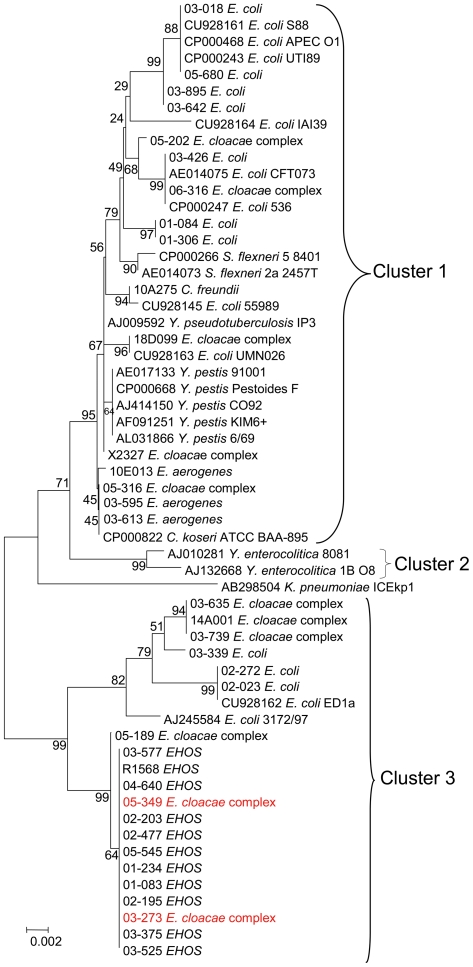
Phylogenetic tree based on 58 *intB* sequences. The phylogenetic tree is based on a neighbor-joining algorithm and 10,000 bootstrap iterations with Mega4.0 on *intB* sequences, showing the relationships among the 58 *intB* sequences included in this study. The scale bar represents a 1% difference in nucleotide sequence. Red: ECC isolates with identical *intB* sequences and otherwise genotyped as the EHOS. The tree is divided into three separate branches with high bootstrap values except for *Klebsiella pneumoniae* ICEkp1, which demonstrated low bootstrap values with other isolates, suggesting it is a separate cluster.

The *intB* gene is genetically the most diverse in the analyzed region of the HPI. However, this did not affect the data. When the *intB* gene was excluded from the HPI analysis, results were similar (Figure S6A). If only the *intB* sequence is used for this set of 23 isolates, the results are almost congruent with the results from the conserved region of the HPI (Figure S6B). Therefore, only the *intB* gene sequences were used for genetic comparison. Since our aim was to determine if the HPI of the EHOS was closely genetically related to other described HPIs, *intB* was a reasonable alternative to compare many isolates. The *intB* genes of 40 *irp2*-positive isolates were sequenced. Four isolates contained a truncated *intB* gene. Alignment with complete *intB* genes demonstrated that in these four isolates, the *intB* genes lacked base pairs 124 to 470. Therefore, these four *intB* sequences were excluded from analysis. The data of the remaining 36 *intB* sequences were combined with 22 sequences of *intB* present in GenBank to generate a phylogenetic tree (Figure 5). Neutrality tests (Taijma's D test and Fu's F test) were not significant for positive selection (both p>0.1), indicating a neutral selection process for the *intB* gene. This indicates that *intB* sequences are suitable for phylogenetic studies since the *intB* gene is only affected by random mutations. Three clusters with >90% bootstrap values were evident. One *intB* sequence of a *K. pneumoniae* isolate with a low bootstrap value differed from all other isolates and fell outside the three clusters. All *Enterobacteriaceae* with cluster 3 type *intB* sequences (three *E. coli*, six ECC and 11 EHOS, Figure 5) contained a sequence (partly) similar to the HPI-ICEs of EHOS and ECOR31. Furthermore, one ECC isolate with a cluster 1 *intB* and all three *E. aerogenes* isolates also had a sequence (partly) similar to the HPI-ICEs of EHOS and ECOR31. The other isolates with a cluster 1 *intB* did not contain genes of the HPI-ICE.

**Figure 6:**
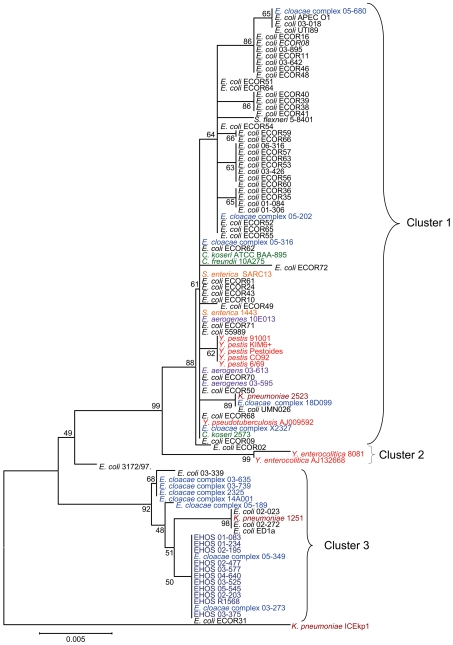
Phylogenetic tree based on 96 792-bp fragment of *intB* sequences. Phylogenetic tree based on a neighbor-joining algorithm and 10,000 bootstrap iterations with Mega4.0 for a 792-bp fragment of the *intB* gene that shows the relationships among the 96 *intB* sequences included in this study. The scale bar represents a 1% difference in nucleotide sequence. Each genus is depicted with a different color. The tree is divided into three separate braches with high bootstrap values except for *Klebsiella pneumoniae* ICEkp1 and *E. coli* 3172/97, which demonstrated low bootstrap values with the other isolates, suggesting separate clusters or recombination of the gene. Cluster 1 contains 68 isolates of nine different species; cluster 2 contains two *Y. enterocolitica* species; and cluster 3 contains five *E. coli* strains, seven ECC strains, eleven EHOS strains and one *K. pneumoniae* strain, in which two ECCs, all EHOSs and *E. coli* ECOR31 are identical.

All eleven EHOS isolates had identical *intB* sequences, showing that the mutation rate in the *intB* gene is low enough to study relatedness of HPIs with other isolates. As shown in Figure 5, there are two ECC isolates (03-273 and 05-349) with the same *intB* gene. Moreover, these isolates also had the genomic island and the three other genetic modules located in the same orientation as the EHOS. These results provide further evidence for combinatorial transfers of the HPI and the other transferable genetic elements. More extensive analysis was possible with a smaller fragment. This previously analyzed 792 bp *intB* gene fragment was used to compare *intB* sequences from one *C. freundii* isolate, two *C. koseri* isolates, eleven EHOS isolates, three *E. aerogenes* isolates, twelve ECC isolates (not EHOS), fifty-four *E. coli* isolates, three *K. pneumoniae* isolates, two *Salmonella enterica* isolates, one *Shigella flexneri* isolates, two *Y. enterocolitica* isolates, five *Y. pestis* isolates, and one *Y. pseudotuberculosis* isolates. The three clusters in the tree based on the complete *intB* gene were also found in the tree constructed with 97 isolates using only 792 bp of the *intB* gene (Figure 6). All isolates used for the analysis of the complete *intB* gene clustered similarly, except for *E. coli* 3172/97, which formed a separate branch with a low bootstrap value. *K. pneumoniae* NTUHK2004 (identical in sequence to *K. pneumoniae* ICE*Kp1*) formed a separate branch with a low bootstrap value, similarly to the phylogenetic tree based on the complete *intB* gene (Figure 5). The *intB* gene fragment of the EHOS and two other ECC isolates (03-273 and 05-349) were identical with the *intB* fragment of ECOR31. Furthermore, this cluster 3 also contained four other *E. coli* isolates, five other ECC isolates and one *K. pneumoniae* isolate. Two *Y. enterocolitica* isolates formed a separate branch (cluster 2). The remaining 68 isolates are all located in cluster 1, which contains nine different species.

### Expression of the HPI Iron Uptake System

Yersiniabactin production was tested in 41 of the 44 HPI-positive isolates used for ICE characterization with a GFP-reporter assay. Included were twelve ECC (not EHOS) isolates, fourteen EHOS isolates, ten *E. coli* isolates, three *E. aerogenes* isolates, and two *C. freundii* isolates (Table S2). Five HPI-negative ECC isolates were used as negative controls.

All 26 HPI-positive ECCs, including 14 EHOS isolates, two *C. freundii* isolates, eight *E. coli* isolates, and one *E. aerogenes* isolate tested positive for yersiniabactin production. Two *E. coli* isolates, two *E. aerogenes* isolates and the negative controls were negative (Table S2).

The expression of HMWP1 and HMWP2 was tested in 14 isolates by SDS-PAGE. Four pairs of EHOS isolates with and without HPI were selected, as were four HPI-positive ECC isolates belonging to different PFGE types and two HPI-positive *E. aerogenes* strains.

All tested HPI-positive isolates were able to produce both HMWP1 and HMWP2, but only under iron-depleted conditions (data not shown). The putative HMWP2 band from one EHOS was confirmed by Edman degradation of the first eight amino acids (data not shown). No HMWP1 or HMWP2 was detected in the HPI-negative isolates or during growth in the presence of iron.

The HMWP2 of the two *E. aerogenes* isolates, which were negative in the GFP assay, were approximately 10 kDa larger than expected; possibly HMWP2 in these two *E. aerogenes* was dysfunctional, which may explain the negative GFP-assay test result (data not shown).

## Discussion

The results of this study show that the *E. hormaechei* outbreak strain (EHOS) responsible for a nationwide outbreak in The Netherlands contained an HPI with a functional yersiniabactin-iron-uptake system, enabling the strain to obtain iron in environments with very limited free iron such as the human body. There was circumstantial evidence that the HPI-ICE*Eh1* was transferred *in vivo*. The EHOS contained a new HPI variant, now termed HPI-ICE*Eh1*, with an ICE element highly similar to the ICE element of HPI-ICE*Ec1* from *E. coli* ECOR31 [7], [10]. The iron-uptake system of HPI-ICE*Eh1* was expressed, as has been previously described for other HPIs [9], [45]. Whether the HPI contributed to the epidemic behavior of the EHOS cannot be concluded from these data. In several *Enterobacteriacae* it has been shown that HPI-containing isolates are more virulent than isolates lacking this island [7], [9], [21]–[24]. Possibly, the increased pathogenicity increases the severity of the infections and consequently the bacterial load in patients, which possibly also increases their length of hospital stay. Both of these factors will increase the chance of cross-transmission to other patients or to the environment. Another explanation could be that optimal iron acquisition is essential for colonization of heavily colonized habitats such as the colon [46]. The ‘normal’ habitat of *E. hormaechei* is speculative because most often isolates of the *E. cloacae* complex isolates are not further determined than the level of *E. cloacae*. Recent studies showed that most infections in humans with ECC are predominantly caused by *E. hormaechei* [13], [47] while the relative presence of *E. hormaechei* compared to ECC isolates in the gut was low [13]. *E. hormaechei* has also been isolated from animals and occasionally drinking water reservoirs [47], [48]. In addition, *E. hormaechei* was isolated during selection studies searching for rhamnolipid-producing strains from a biodiesel facility, the search for selenium reducing bacteria from a coal mine tailing pond sediment, and the examination of salinated soil where the rhizosphere of wheat was grown [49]–[51]. This indicates that *E. hormaechei* is widely distributed in the environment and capable to adapt to different niches.

Analysis of the genome of an EHOS showed that the EHOS contained multiple integrations of mobile elements. Due to the putative presence of two different genomic islands together with the three putative genomic modules and the demonstrated flexibility of excision and putative integration, this part of the *E. hormaechei* genome may be considered an integration hotspot (IHS). This IHS increases the genomic plasticity of the bacterium. Except for HPI-ICE*Eh1*, the functions of the genes located on the genomic islands in the IHS are largely unknown. *Eh*GI1 may be involved in iron uptake similarly to the HPI of HPI-ICE*Eh1*. Genes located on genomic modules three, four, and five are possibly involved in regulation and transport. Although speculative, the presence of AcrA and AcrB-like protein encoding genes indicate that a tripartite efflux pump is present. In *Enterobacteriaceae*, these types of efflux pumps extrude cytotoxic substances from the cell directly into the medium, bypassing the periplasm and thereby reducing susceptibility to toxic compounds [52].

One putatively truncated integrase in *Eh*GI1 (*intB2*) and an integrase in HPI-ICE*Eh1* (*intB*) were present and had a sequence similarity of 95%. Most likely, the integrase of HPI-ICE*Eh1* is functional, because several different combinations of genomic islands and genomic modules were excised from the chromosome and formed circular structures, the integrase of *Eh*GI1 was truncated, and *Eh*GM3, *Eh*GM4, and *Eh*GM5 lack integrase genes. Therefore, we speculate that *Eh*GI1 and the *Eh*GMs are co-transferred by HPI-ICE*Eh1* or are remnants from previous recombination events. Recent studies showed the transfer of HPI-ICE*Kp1* to *E. coli* and *K. pneumoniae* in a conjugation experiment and possibly *in vivo* to other *K. pneumoniae* isolates [7]. Another study showed horizontal transfer of the HPI within *E. coli* species [11]. Here we provide evidence that multiple combinatorial transfers of the HPI and other genetic modules between ECC isolates most likely occurred. The idea of multiple combinatorial transfers of genomic islands and modules is supported by the following results. Two genomic islands (*Eh*GI1 and HPI-ICE*Eh1*) and three genomic modules (*Eh*GM3, *Eh*GM4, and *Eh*GM5) were detected in two other ECC isolates with different genotypes in the same order. In addition, the *intB* gene sequences were identical, between the different *intB* genes of the EHOS and the two ECC isolates that have different genotypes whereas this gene shows variability. If this was not a relatively recent event, it would be expected that mutations in *intB* would have occurred. Therefore, it is reasonable to presume that transfers of these elements occurred relatively recently, but certainly before the diversification of the ECC species. Finally, genes encoded by the genomic islands and modules were present in different isolates, indicating transfer of those elements. Transfer of the genetic islands and modules was further supported by the finding that structures highly similar to *Eh*GI1 and *Eh*GI2 and partially similar to *Eh*GM3, *Eh*GM4, and *Eh*GM5 were present in other *Enterobacter* species. Furthermore, there was a significantly higher prevalence (p<0.05) of HPIs in the non-EHOS isolates that were isolated during the EHOS outbreak period in the UMCU in comparison with those not linked to the UMCU EHOS outbreak, which could indicate transfer, although it cannot be excluded that these isolates were selected under the selective pressure in the hospital. All cluster 3 *intB-*containing isolates (partly) had an ICE segment, while only a fraction of the cluster 1 *intB-*containing isolates (partly) harbored an ICE segment, which indicates that the ICE is derived from isolates with the cluster 3 *intB* gene. This conclusion is further supported by the composition of the conserved segments of the HPI in different isolates. Moreover, all isolates with similar ICE elements (except one) clustered in the same branch, which indicates multiple transfer events of the HPI-ICE to other species. Furthermore, the HPI-negative isolates did not contain the ICE segment (or even part of it), supporting the hypothesis that an HPI-ICE is the progenitor of the HPI in *Yersinia* species [10]. The massive combinatorial transfer of the HPI-ICE*Eh1* and other genomic modules between *Enterobacteriaceae* is worrisome. It has been hypothesized that the majority of disease-causing bacteria from the intestine (e.g., *Shigella* spp. or *Yersinia* spp.) may have been derived from commensals that have acquired genes from foreign sources turning them into pathogens [53]. Incorporation of the HPI, or other genomic islands or modules, into chromosomes of other isolates may generate new pathogenic strains, which due to their different genetic backgrounds, may emerge in new niches [3]. The uptake of multiple genomic islands and modules and resistance determinants is in agreement with the hypothesis of genetic capitalism, which states that successful integration and selection of a foreign genetic element increases the number of possible genetic transfer events in the future [17]. The acquisition of the HPI, other genomic islands and multidrug resistance genes by the EHOS underscores this hypothesis [17].

## Supporting Information

Figure S1 Overview of excision and subsequent circularization of *Eh*GI1 and HPI-ICE*Eh1*. A) Schematic presentation of the region in the IHS where excision takes place. Black arrows depict the location and orientation of the primers for the first PCR. Red arrows depict the location of the primers for the nested PCR. B) Schematic presentation of the circular structure formed. C) The circularized fragment generated a product of 2,572 bp. The primers used were ORF77-2F and ORF7-2R (Table S1). The product was analyzed on a 1% agarose gel. D) The circularized fragment generated a product of 1,204 bp from a nested PCR. The product was amplified with the primers ORF77-2F and intB-N1 (Table S1). The product was analyzed on a 1% agarose gel. E) Box W: Schematic presentation of the PCR, nested PCR and sequenced fragment (Accession no: GU086403).
(7.75 MB TIF)

Figure S2 Overview of excision and subsequent circularization of *Eh*GI1, HPI-ICE*Eh1*, and *Eh*GM3. A) Schematic presentation of the region in the IHS where excision takes place. Black arrows depict the location and orientation of the primers for the first PCR. Red arrows depict the location of the primers for the nested PCR. B) Schematic presentation of the circular structure formed. C) The circularized fragment generated a product of 2,046 bp. The primers used were ORF85-2F and ORF7-2R (Table S1). the product was analyzed on a 1% agarose gel. D) The circularized fragment generated a product of 335 bp with a nested PCR. The product was amplified with the primers tRNA-N3 and intB-N1 (Table S1). The product was analyzed on a 1% agarose gel. E) Box X: Schematic presentation of the PCR, nested PCR and sequenced fragment. ^a^Sequencing of the first PCR product with primer ORF7-2R shows 100% homology with the sequence of FN297818. ^b^Sequencing of the first PCR product with primer tRNA-N3 (Accession no: FN556610).
(8.80 MB TIF)

Figure S3 Overview of circularization of HPI-ICE*Eh1*. A) Schematic presentation of the circular structure of HPI-ICEEh1 that is formed. B) The product amplified from the circularized fragment. This was too small and most likely an artifact, as the expected product size from the fragment was 2,651 bp. The primers used were ORF77-2F and YbtS-R (Table S1). The product was analyzed on a 1% agarose gel. C) The circularized fragment generated a product of 1,205 bp with a nested PCR. The product was amplified with the primers ORF77-2F and intB-N1 (Table S1). The product was analyzed on a 1% agarose gel. D) Box Y: Schematic presentation of the PCR, nested PCR and sequenced fragment, (Accession no: FN556611).
(8.35 MB TIF)

Figure S4 Overview of excision and circularization of HPI-ICE*Eh1* and *Eh*GM3. A) Schematic presentation of the region in the IHS that is excised and subsequently forms a circular structure. Black arrows depict the schematic location and orientation of the primers used for the first PCR. Red arrows depict the location of the primers for the nested PCR. B) Schematic presentation of the circular structure formed. C) The circularized fragment generated a product of 2,225 bp. The primers used were ORF85-2F and YbtS-R (Table S1). The product was analyzed on a 1% agarose gel. D) The circularized fragment generated a product of 335 bp with a nested PCR. The product was amplified with the primers tRNA-N3 and intB-N1 (Table S1). The product was analyzed on a 1% agarose gel. E) Box Z: Schematic presentation of the PCR, nested PCR and sequenced fragment (Accession no: FN556612).
(8.92 MB TIF)

Figure S5 Amplified fragments overlapping the junction between the chromosome and the IHS. A) PCR amplification results from EHOS 05-545 with the different primer mixes as presented in panel C. B) PCR amplification results from the HPI-negative isolate EHOS 03-638 with the different primer mixes (panel C). ^a^Non-specific amplified product of the overlapping region between the chromosome and the *intB* or *intB2* gene. ^b^Fragment after excision of *Eh*GI1 and HPI-ICE*Eh1*, showing that *Eh*GM3, *Eh*GM4 and *Eh*GM5 are still part of the chromosome. The junction in 05-545 was sequenced to confirm the results (Accession no: FN556613). ^c^Possible product indicating that occasionally only *Eh*GM4 and *Eh*GM5 are still present in the chromosome.
(5.35 MB TIF)

Figure S6 Phylogenetic analysis of HPI-ICE. A) Phylogenetic tree based on the sequence of the conserved part of the HPI-ICE minus the *intB* gene (homologous to bp 43,852-72,874 of accession no. FN297818) of 23 *Enterobacteriaceae* clustered with Clonalframe. Numbers indicate confidence values of the branches. B) Phylogenetic tree based on the sequence of the *intB* gene (homologous to bp 42,589-43,851 of accession no. FN297818) of 23 *Enterobacteriaceae* used to compare the conserved part of the HPI-ICE clustered with Clonalframe. Numbers indicate confidence values of the branches.
(6.58 MB TIF)

Table S1 Primers used for amplification and sequencing
(0.04 MB XLS)

Table S2 Genes, open reading frames (orfs) and DNA sequences known to be located on genomic islands and modules of the EHOS. “+” represents that a product was amplified and “−” represents a negative result for the different DNA fragments investigated. ^a^Isolated group, 1 isolates of UMCU-ECC group, 2 isolates non-UMCU-ECC group, 3 isolates of the third group, which are isolates that not fulfill the criteria of the first two groups but are HPI-positive.^b^Fragment size 1786 bp; orf20 present, ^c^Fragment size 631 bp; orf20 absent.^d^ECC with the same *intB* gene as the EHOS and also similar genetic modules located in the same orieantation and sequens as EHOS.^e^Deletion in *intB* gene of 347 basepairs.
(0.07 MB XLS)
