# Supplementary figures and images for: Evolution in Quantum Leaps: Multiple Combinatorial Transfers of HPI and Other Genetic Modules in Enterobacteriaceae
Source: PLoS One. 2010 Jan 13;5(1):e8662. doi: 10.1371/journal.pone.0008662 (PMC2801613; doi:10.1371/journal.pone.0008662)

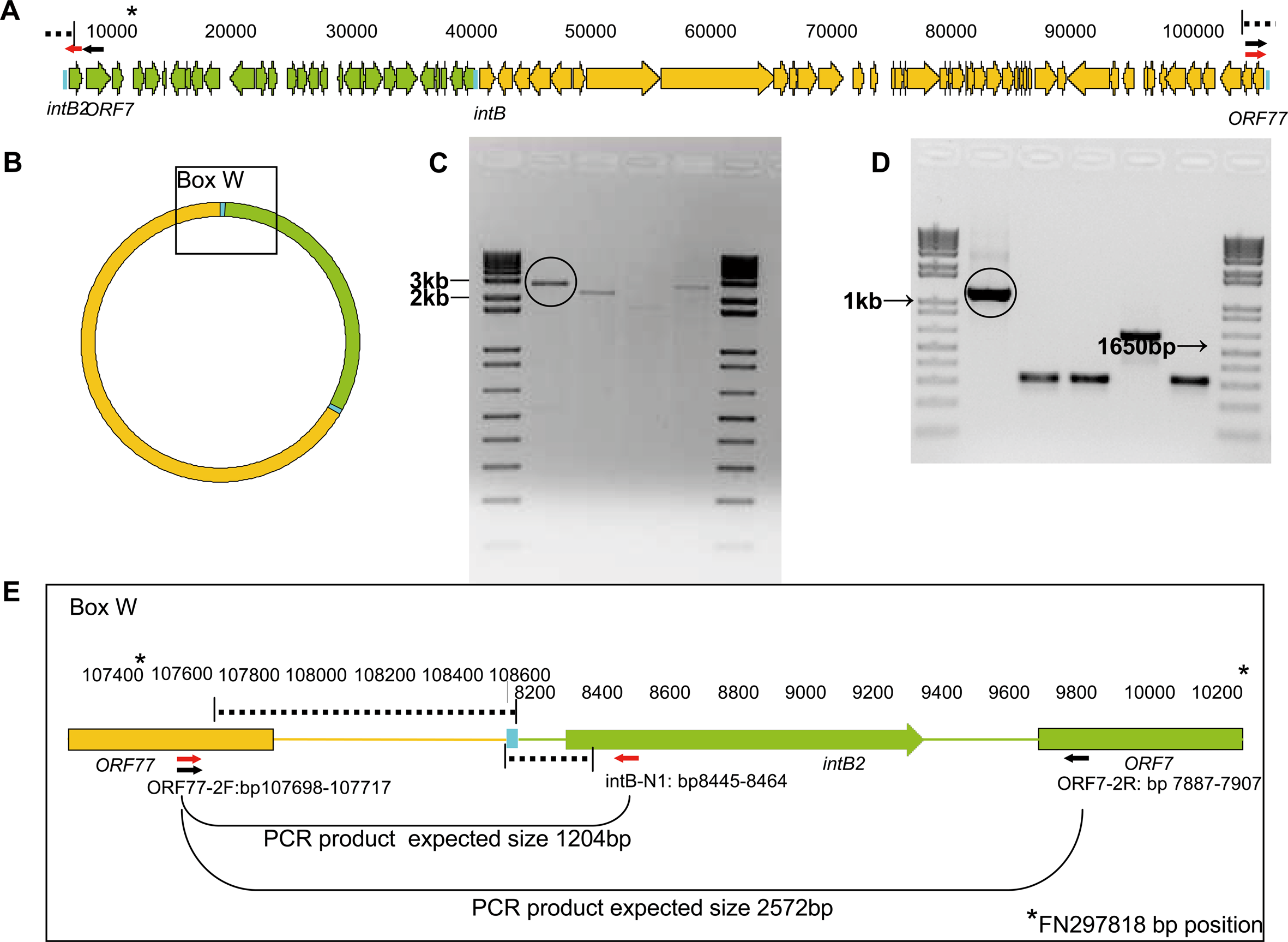

Supplement: Figure S1 — Overview of excision and subsequent circularization of EhGI1 and HPI-ICEEh1. A) Schematic presentation of the region in the IHS where excision takes place. Black arrows depict the location and orientation of the primers for the first PCR. Red arrows depict the location of the primers for the nested PCR. B) Schematic presentation of the circular structure formed. C) The circularized fragment generated a product of 2,572 bp. The primers used were ORF77-2F and ORF7-2R (Table S1). The product was analyzed on a 1% agarose gel. D) The circularized fragment generated a product of 1,204 bp from a nested PCR. The product was amplified with the primers ORF77-2F and intB-N1 (Table S1). The product was analyzed on a 1% agarose gel. E) Box W: Schematic presentation of the PCR, nested PCR and sequenced fragment (Accession no: GU086403). (7.75 MB TIF) [file pone.0008662.s001.tif]

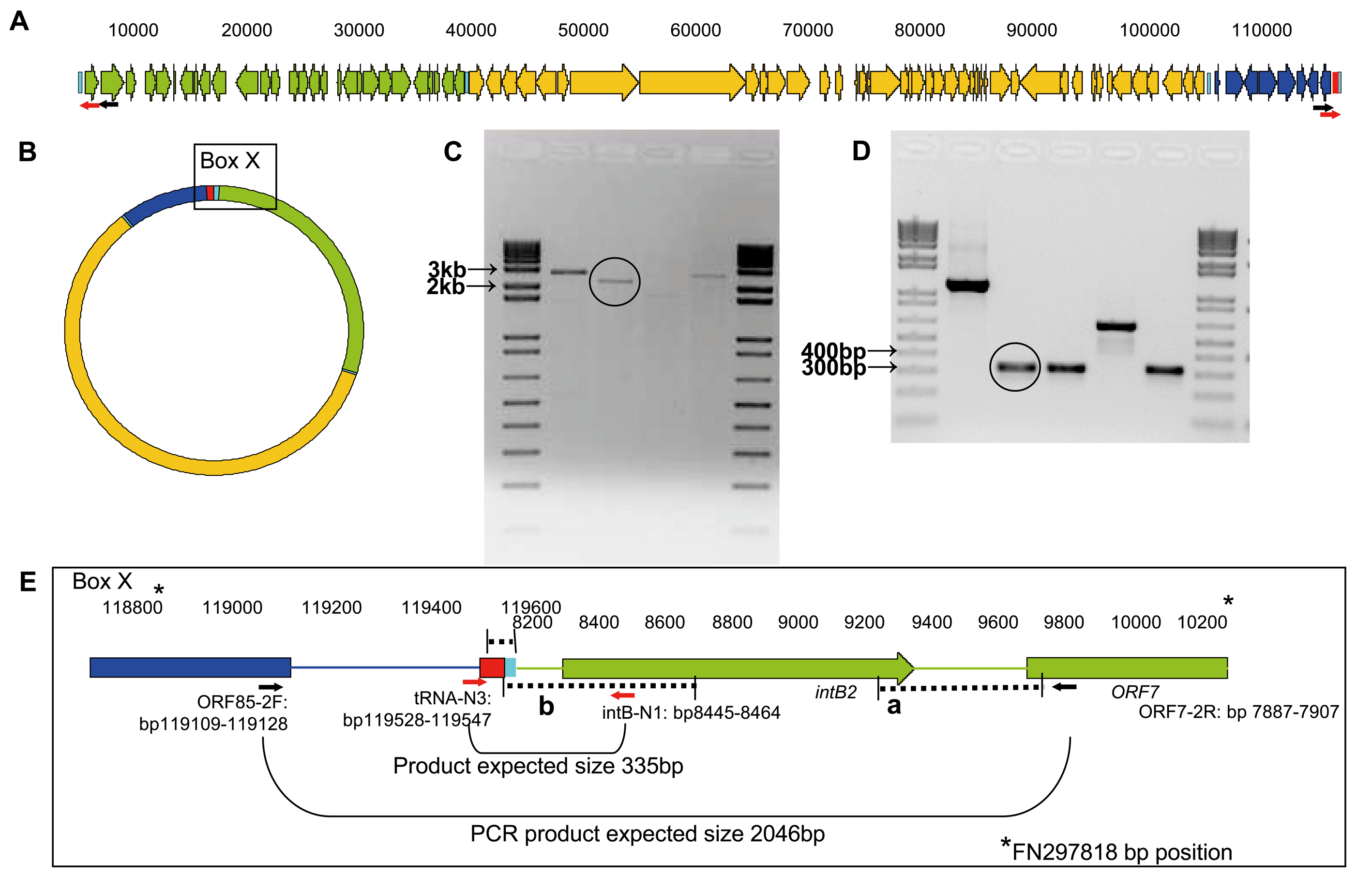

Supplement: Figure S2 — Overview of excision and subsequent circularization of EhGI1, HPI-ICEEh1, and EhGM3. A) Schematic presentation of the region in the IHS where excision takes place. Black arrows depict the location and orientation of the primers for the first PCR. Red arrows depict the location of the primers for the nested PCR. B) Schematic presentation of the circular structure formed. C) The circularized fragment generated a product of 2,046 bp. The primers used were ORF85-2F and ORF7-2R (Table S1). the product was analyzed on a 1% agarose gel. D) The circularized fragment generated a product of 335 bp with a nested PCR. The product was amplified with the primers tRNA-N3 and intB-N1 (Table S1). The product was analyzed on a 1% agarose gel. E) Box X: Schematic presentation of the PCR, nested PCR and sequenced fragment. aSequencing of the first PCR product with primer ORF7-2R shows 100% homology with the sequence of FN297818. bSequencing of the first PCR product with primer tRNA-N3 (Accession no: FN556610). (8.80 MB TIF) [file pone.0008662.s002.tif]

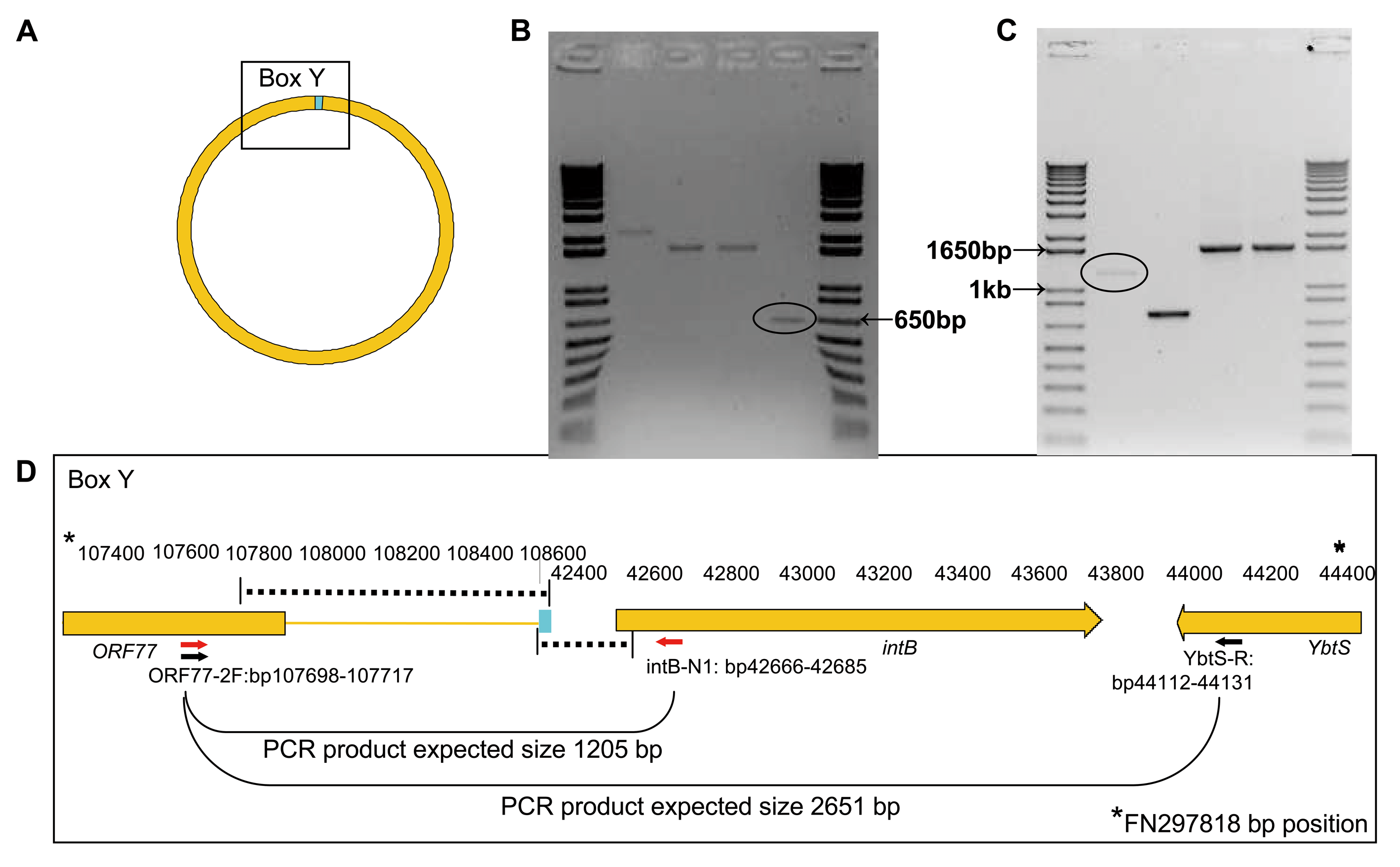

Supplement: Figure S3 — Overview of circularization of HPI-ICEEh1. A) Schematic presentation of the circular structure of HPI-ICEEh1 that is formed. B) The product amplified from the circularized fragment. This was too small and most likely an artifact, as the expected product size from the fragment was 2,651 bp. The primers used were ORF77-2F and YbtS-R (Table S1). The product was analyzed on a 1% agarose gel. C) The circularized fragment generated a product of 1,205 bp with a nested PCR. The product was amplified with the primers ORF77-2F and intB-N1 (Table S1). The product was analyzed on a 1% agarose gel. D) Box Y: Schematic presentation of the PCR, nested PCR and sequenced fragment, (Accession no: FN556611). (8.35 MB TIF) [file pone.0008662.s003.tif]

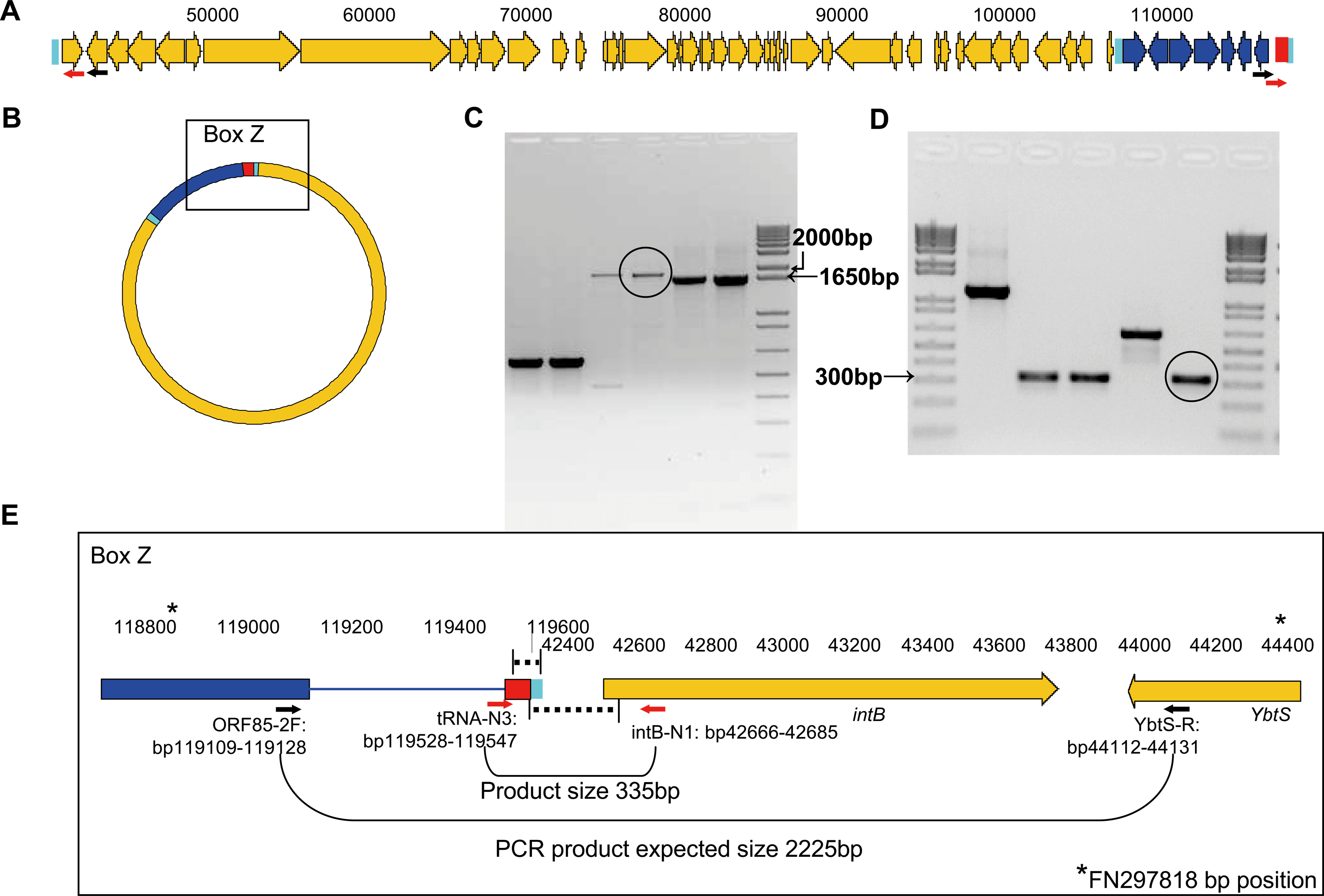

Supplement: Figure S4 — Overview of excision and circularization of HPI-ICEEh1 and EhGM3. A) Schematic presentation of the region in the IHS that is excised and subsequently forms a circular structure. Black arrows depict the schematic location and orientation of the primers used for the first PCR. Red arrows depict the location of the primers for the nested PCR. B) Schematic presentation of the circular structure formed. C) The circularized fragment generated a product of 2,225 bp. The primers used were ORF85-2F and YbtS-R (Table S1). The product was analyzed on a 1% agarose gel. D) The circularized fragment generated a product of 335 bp with a nested PCR. The product was amplified with the primers tRNA-N3 and intB-N1 (Table S1). The product was analyzed on a 1% agarose gel. E) Box Z: Schematic presentation of the PCR, nested PCR and sequenced fragment (Accession no: FN556612). (8.92 MB TIF) [file pone.0008662.s004.tif]

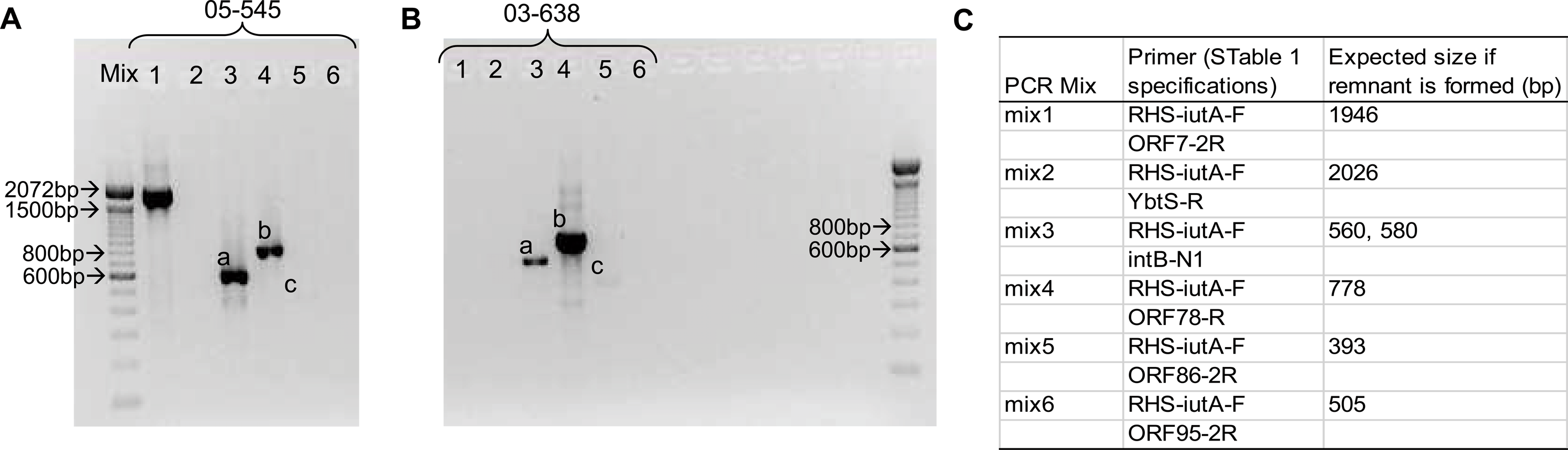

Supplement: Figure S5 — Amplified fragments overlapping the junction between the chromosome and the IHS. A) PCR amplification results from EHOS 05-545 with the different primer mixes as presented in panel C. B) PCR amplification results from the HPI-negative isolate EHOS 03-638 with the different primer mixes (panel C). aNon-specific amplified product of the overlapping region between the chromosome and the intB or intB2 gene. bFragment after excision of EhGI1 and HPI-ICEEh1, showing that EhGM3, EhGM4 and EhGM5 are still part of the chromosome. The junction in 05-545 was sequenced to confirm the results (Accession no: FN556613). cPossible product indicating that occasionally only EhGM4 and EhGM5 are still present in the chromosome. (5.35 MB TIF) [file pone.0008662.s005.tif]

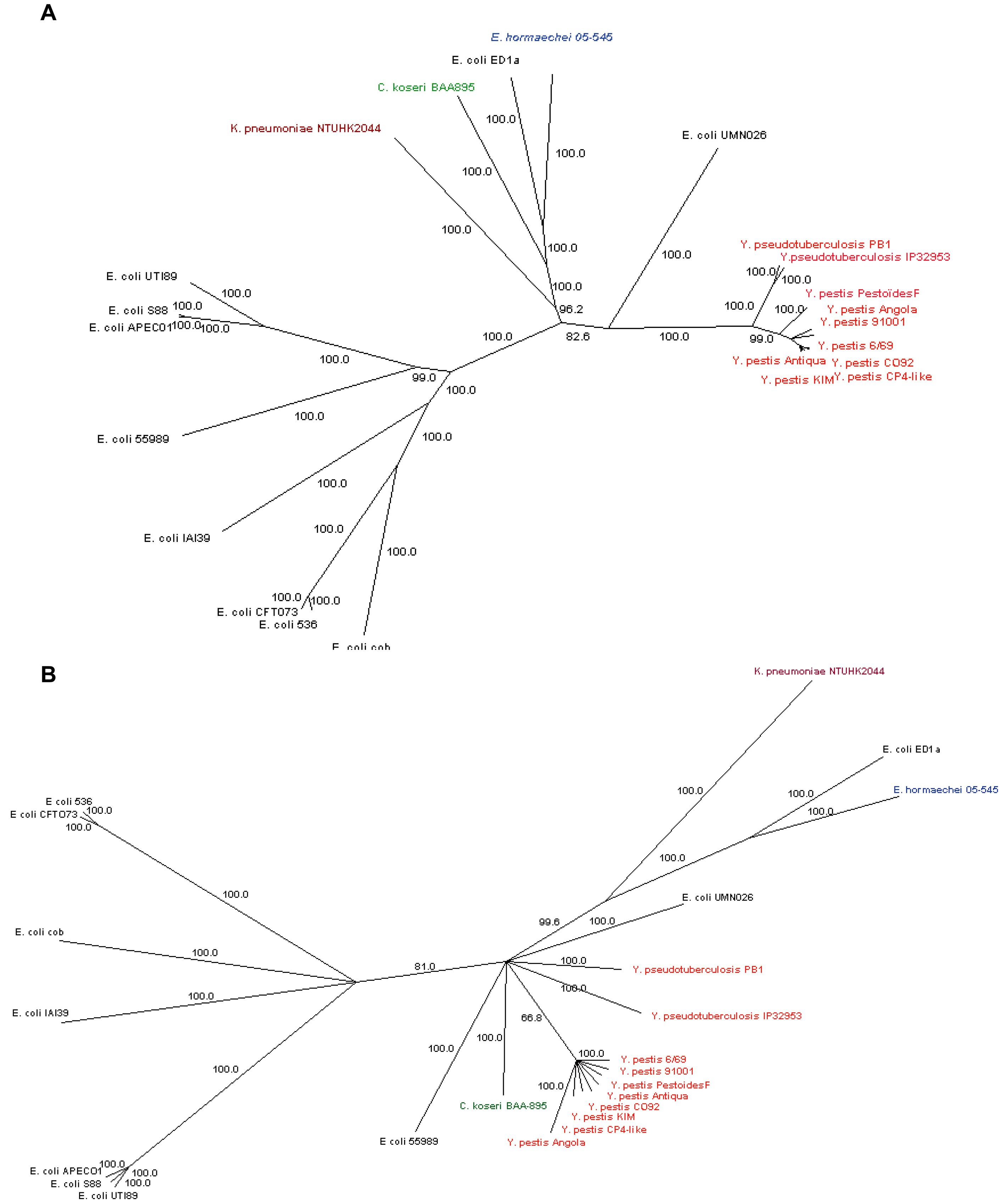

Supplement: Figure S6 — Phylogenetic analysis of HPI-ICE. A) Phylogenetic tree based on the sequence of the conserved part of the HPI-ICE minus the intB gene (homologous to bp 43,852-72,874 of accession no. FN297818) of 23 Enterobacteriaceae clustered with Clonalframe. Numbers indicate confidence values of the branches. B) Phylogenetic tree based on the sequence of the intB gene (homologous to bp 42,589-43,851 of accession no. FN297818) of 23 Enterobacteriaceae used to compare the conserved part of the HPI-ICE clustered with Clonalframe. Numbers indicate confidence values of the branches. (6.58 MB TIF) [file pone.0008662.s006.tif]
